# *Pistacia vera* L. oleoresin and levofloxacin is a synergistic combination against resistant *Helicobacter pylori* strains

**DOI:** 10.1038/s41598-019-40991-y

**Published:** 2019-03-15

**Authors:** Silvia Di Lodovico, Edoardo Napoli, Emanuela Di Campli, Paola Di Fermo, Davide Gentile, Giuseppe Ruberto, Antonia Nostro, Emanuela Marini, Luigina Cellini, Mara Di Giulio

**Affiliations:** 1 Department of Pharmacy, “G. d’Annunzio” University Chieti-Pescara, Chieti, Italy; 2 Institute of Biomolecular Chemistry, National Research Council ICB-CNR, Catania, Italy; 3 Department of Chemical, Biological, Pharmaceutical and Environmental Sciences, University of Messina, Messina, Italy; 4 Unit of Microbiology, Department of Biomedical Sciences and Public Health, Polytechnic University of Marche, Ancona, Italy

## Abstract

The increasing multidrug resistance in *Helicobacter pylori*, also correlated to its biofilm‐forming ability, underlines the need to search novel strategies to improve the eradication rate. Natural compounds are proposed as antibiotic-resistant-breakers capable to restore the efficacy of conventional drugs. Aim of this work was to evaluate the capability of *Pistacia vera* L. oleoresin (ORS) to synergize with levofloxacin (LVX) against resistant *H*. *pylori* strains. The antimicrobial activity of *P*. *vera* L. ORS and LVX and their combinations was determined by MIC/MBC (in neutral and acidic environments) and checkerboard tests. The anti-biofilm effect was determined by biomass quantification. *In vivo Galleria mellonella* model was used to confirm *in vitro* data. *Pistacia vera* L. ORS and LVX MICs ranged respectively from 780 to 3120 mg/l and from 0.12 to 2.00 mg/l, at pH 7.0 and 5.5. MBCs were similar to MICs. *Pistacia vera* L. ORS was able to synergize with LVX, restoring its effectiveness in LVX resistant strains. *Pistacia vera* L. ORS, LVX and their synergistic combinations displayed significant biofilm reduction. *Pistacia vera* L. ORS and LVX, showed protective effect against *H*. *pylori* infection on *G*. *mellonella* (62% and 63% of survival, respectively). *Pistacia vera* L. ORS can be considered a promising potentiator to restore the effectiveness of LVX tackling the *H*. *pylori* antibiotic resistance phenomenon.

## Introduction

*Helicobacter pylori* is a gastroduodenal pathogen that plays an important role in the pathogenesis of chronic gastritis, peptic ulcer, gastric adenocarcinoma and MALT (mucosa-associated lymphoid tissue) lymphoma. *H*. *pylori* infection is difficult to eradicate and requires the combination of different drugs such as clarithromycin, levofloxacin (LVX), amoxicillin, metronidazole, tetracycline and proton pump inhibitor^1–3^. The increase of antimicrobial resistance and the failure of therapeutic regimens, strongly underline the need to find novel strategies to enhance the eradication rate, also considering the capability of *H*. *pylori* to grow in biofilm mode^4–6^. Moreover, the *H*. *pylori* antimicrobial resistance profiles vary in different geographic areas, therefore the selection of therapeutic regimens needs to be adjusted according to local resistance patterns, if available^7–10^. For all these reasons, the search for alternative and effective new therapeutic schemes is important and urgent^11–14^.

New approaches to tackle the infections related to multidrug resistant (MDR) bacteria are recently proposed in the search for antibiotic-resistance-breakers capable to synergize with conventional drugs restoring their effectiveness^15–17^.

To this regards, much interest has been revived in the study of antimicrobial/antivirulence effects of formulations based on medicinal plants^15,18,19^. Several plants produce a variety of secondary metabolites such as phenolics, terpenoids and alkaloids, which possess a wide spectrum of biological activities, including the antibacterial ones^14,19–21^. They interact with the lipidic bilayer of the cytoplasmic membrane, membrane proteins and enzymes involved in the synthesis of macromolecules, causing increased permeability, loss of proton-motive force and cellular material^14^.

The plants of the genus *Pistacia* (Anacardiaceae family) are widely cultivated in Mediterranean countries and comprise over 600 species; two of them, *P*. *lentiscus* L. (known as ‘mastic’) and *P*. *vera* L., are the commonly cultivated species; while the other species are mostly used as rootstock for *P*. *vera* L.^18^. The main *Pistacia* plants products are the fruits, those of *P*. *vera* L. are edible and so-called “green-gold” for their high value as dried fruit, while those from *P*. *lentiscus* L. are used since ancient time to produce *lentiscus* oil for dietary and folk-medicine purposes. *Pistacia* plants are able to produce an oleoresin (ORS) which can be extracted from incisions made in the tree trunk. In particular, the resin of *P*. *lentiscus* L. var. *Chia* (mastic gum) has been used for more than 2500 years in traditional Greek medicine for treating several diseases mainly gastrointestinal disorders, relief of abdominal discomfort, gastralgia, dyspepsia and peptic ulcer^22,23^. It has also been used as a masticatory to prevent oral plaque. Mastic gum has been reported to be effective in the treatment of benign gastric ulcers and duodenal ulcers and for *H*. *pylori* infection^22–24^.

Many studies demonstrated that plant components can act in synergy with several antibiotics against antibiotic-resistant pathogens, including *H*. *pylori*, improving the eradication rate^13,15,25,26^.

The *H*. *pylori* resistance to antimicrobials commonly used in therapy has increased in the last years and, in particular, the resistance to LVX is a worrying phenomenon that can explain the failure of therapies used up to now. In an our *in vitro* and *in vivo* study^27^, the addition of a natural compound to traditional therapeutic schemes, enhances the effect of LVX by reducing the level of bacterial resistance.

Based on these considerations, the aim of the present study was to evaluate the antimicrobial and anti-biofilm activities of *P*. *vera* L. ORS alone and combined to LVX against resistant strains of *H*. *pylori*. The evaluation of the chemical fingerprinting of the major acidic and neutral *P*. *vera* L. ORS fractions was performed. All the detected *in vitro* antimicrobial data were also confirmed *in vivo* by using *Galleria mellonella* model that is a recognized experimental assay for *H*. *pylori* infection.

## Results

The neutral and acidic fractions of *P*. *vera* L. ORS were analyzed by Gas-chromatograph Mass Spectrometry (GS-MS). The neutral fraction contained a considerable amount of monoterpenes (27% both hydrocarbons and oxygenated) and a higher percentage of neutral triterpenes (59%). The acidic fraction had no monoterpenes and showed a higher percentage of acid triterpenes up to 69.7%. Mass spectra comparison allowed the identification of triterpenes, belonging to the 12- and 18-unsaturated oleanenes/ursenes, dammaranes and tirucallene derivative chemical families. The main compounds detected in *P*. *vera* L. ORS were hydroxydammarenone, tirucallol, isomasticadienoninc and masticadienoninc acids.

The antibacterial effect of *P*. *vera* L. ORS and LVX was evaluated against *H*. *pylori* strains to determine the susceptibility both at pH 7.0 and at 5.5 (Table 1). The MIC values of *P*. *vera* L. ORS and LVX ranged from 780 to 3120 mg/l and from 0.12 to 2.00 mg/l, respectively, both in neutral and acid environments. In general, the MBC values of *P*. *vera* L. ORS, against *H*. *pylori* strains, were equal or one step above to the MIC values, except for *H*. *pylori* 9A/12 in which the MBC at pH 7.0 was two step above the MIC. The MBC values of LVX against *H*. *pylori* strains were always coincident to the MIC values, both at pH 7.0 and at 5.5, except for *H*. *pylori* 4A/13 (one step above). In Table 1 also are the best combinations of *P*. *vera* L. ORS and LVX with the values of FIC Index (FIC I) for each detected strain. Synergism was recorded in 31 of 32 strains (FIC I from 0.18 to 0.50) and an additive effect (FIC I = 0.75) was displayed in the reference *H*. *pylori* strain. Antagonism was not recorded. The MICs of LVX for all MDR and resistant strains (listed in Supplementary Table S1) were lower than the respective breakpoints when tested in combination with *P*. *vera* L. ORS with concentrations ranging from 0.03 to 0.25 mg/l (Table 1). In particular, the best FIC I value was recorded for the resistant *H*. *pylori* 11F/11 with value of 0.18 with a MIC reduction of 4- and 2-fold for *P*. *vera* L. ORS and LVX, respectively.

**Table 1:** Minimum Inhibitory Concentration and Minimum Bactericidal Concentration of *P*. *vera* L. ORS and LVX against *H*. *pylori* strains at two pH values and the effect of the combination of *P*. *vera* L. ORS and LVX determined by the checkerboard test and calculation of the Fractional Inhibitory Concentration Index (FIC I)

*H*. *pylori* strains	*P*. *vera* L. ORS (mg/l)				LVX (mg/l)				Best combination ORS + LVX (mg/l)^#^	FIC I*
	MIC		MBC		MIC		MBC			
	pH		pH		pH		pH			
	7.0	5.5	7.0	5.5	7.0	5.5	7.0	5.5		
11F/11	1560	1560	1560	3120	1.00	1.00	1.00	1.00	90 + 0.12	0.18
2A/12	1560	3120	3120	6250	0.50	1.00	0.50	1.00	190 + 0.12	0.36
3F/12	1560	1560	3120	3120	1.00	1.00	1.00	1.00	390 + 0.12	0.37
4A/12	1560	3120	1560	3120	0.50	0.50	0.50	0.50	390 + 0.12	0.49
7A/12	1560	1560	3120	3120	1.00	1.00	1.00	1.00	390 + 0.25	0.50
9A/12	780	1560	3120	3120	1.00	1.00	1.00	1.00	190 + 0.25	0.49
12F/12	3120	3120	3120	3120	1.00	1.00	1.00	1.00	780 + 0.25	0.50
13A/12	1560	3120	1560	3120	0.50	0.50	0.50	0.50	390 + 0.12	0.49
1F/13	1560	1560	3120	3120	1.00	1.00	1.00	1.00	390 + 0.25	0.50
3F/13	780	780	1560	1560	0.50	0.50	0.50	0.50	190 + 0.12	0.48
4A/13	3120	3120	6250	6250	0.50	0.50	1.00	1.00	780 + 0.12	0.49
5A/13	1560	1560	3120	3120	1.00	1.00	1.00	1.00	390 + 0.25	0.50
10A/13	1560	3120	1560	3120	1.00	1.00	1.00	1.00	390 + 0.25	0.50
13A/13	1560	1560	3120	3120	1.00	1.00	1.00	1.00	390 + 0.25	0.50
20A/13	1560	1560	3120	3120	1.00	1.00	1.00	1.00	390 + 0.25	0.50
23A/13	1560	3120	3120	3120	1.00	1.00	1.00	1.00	190 + 0.12	0.24
24F/13	780	1560	1560	1560	1.00	1.00	1.00	1.00	190 + 0.12	0.36
25F/13	1560	1560	3120	3120	1.00	1.00	1.00	1.00	390 + 0.25	0.50
26A/13	3120	3120	6250	6250	2.00	2.00	2.00	2.00	780 + 0.25	0.37
5F/14	3120	3120	6250	6250	1.00	1.00	1.00	1.00	780 + 0.12	0.37
10A/14	1560	1560	6250	6250	0.50	0.50	0.50	0.50	190 + 0.12	0.36
29A/14	1560	1560	3120	3120	1.00	1.00	1.00	1.00	390 + 0.12	0.37
3F/15	780	780	780	1560	1.00	1.00	1.00	1.00	190 + 0.12	0.36
4A/15	1560	1560	3120	3120	1.00	1.00	1.00	1.00	390 + 0.25	0.50
8F/15	1560	1560	1560	1560	0.50	0.50	0.50	0.50	390 + 0.12	0.49
30A/15	1560	1560	1560	3120	0.50	0.50	0.50	0.50	390 + 0.12	0.49
1A/16	1560	1560	3120	3120	1.00	1.00	1.00	1.00	390 + 0.25	0.50
5A/16	1560	1560	1560	1560	1.00	1.00	1.00	1.00	190 + 0.12	0.24
7F/16	3120	3120	3120	3120	0.50	0.50	0.50	0.50	780 + 0.12	0.49
14A/16	3120	3120	3120	3120	1.00	1.00	1.00	1.00	390 + 0.25	0.37
9F/13	1560	1560	3120	3120	0.12	0.12	0.12	0.12	390 + 0.03	0.50
ATCC 43629	3120	3120	6250	6250	0.12	0.12	0.12	0.12	1560 + 0.03	0.75

^*^See Materials and Methods. ^#^Combination of sub-MIC of *P*. *vera* L. ORS and LVX yielding the lowest FIC I, that is the best combination. Synergisms (FIC I ≤ 0.5) were detected in 31 out of 32 strains and an additive effect (FIC I > 0.5–4.0) was displayed in the reference *H*. *pylori* strain. Antagonism was not recorded (FIC I ≥ 4.0).

**Figure 1:**
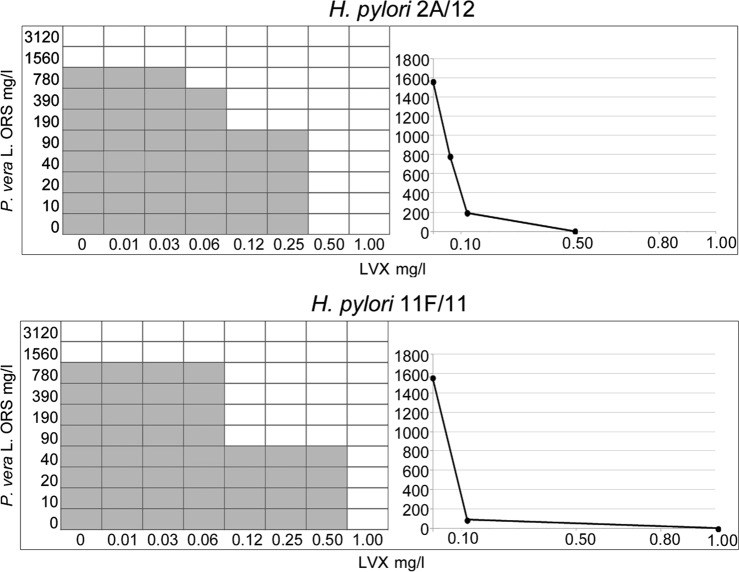
Representative images of checkerboard assays and isobolograms for evaluation of synergism between *P*. *vera* L. ORS and LVX combinations against *H*. *pylori* 2A/12 and *H*. *pylori* 11F/11. The calculation of the best *P*. *vera* L. ORS and LVX combination and the Fractional Inhibitory Concentration Index (FIC I) values were interpreted as follow: synergism (FIC I ≤ 0.5), antagonism (FIC I ≥ 4.0), and additive effect (FIC I > 0.5–4.0). For *H*. *pylori* 2A/12, the MIC values are 1560 mg/l and 0.50 mg/l for *P*. *vera* L. ORS and LVX, respectively. For *H*. *pylori* 11F/11, the MIC values are 1560 mg/l and 1.00 mg/l for *P*. *vera* L. ORS and LVX, respectively. The grey zone represents the bacterial growth and the white zone represents the inhibition of bacterial growth in presence of *P*. *vera* L. ORS and LVX. The synergistic combinations of *P*. *vera* L. ORS and LVX are the white zone in which FIC I is ≤0.5. On the right, the isobolograms illustrate the result of the checkerboard assay and the FIC I values, showing the synergistic curve. The x axis of the isobologram represents the dose of LVX and the y axis represents the dose of *P*. *vera* L. ORS. The imaginary straight line connecting the intercept points represents no interaction. Between this line and the synergistic curve, there is the area of synergistic (FIC I ≤ 0.5) and additive (FIC I > 0.5–4.0) interactions. Values above of the straight line represent antagonistic interactions (FIC I ≥ 4.0).

Figure 1 shows representative images of the checkerboard assays and the isobolograms combining different concentrations of *P*. *vera* L. ORS and LVX against two strains: the MDR *H*. *pylori* 2A/12 and the resistant *H*. *pylori* 11F/11.

*Pistacia vera* L. ORS is able to restore the effectiveness of LVX in resistant *H*. *pylori* strains.

The anti-biofilm effect of *P*. *vera* L. ORS and LVX alone (Table 2) and their synergistic combinations (Table 3), were also evaluated for two MDR, two resistant and two susceptible *H*. *pylori* strains. *Pistacia vera* L. ORS and LVX at sub-MIC concentrations displayed reduction rates ranging from 8.43% ± 1.79 to 93.45% ± 4.34 and from 22.09% ± 2.12 to 91.98% ± 0.38, respectively (Table 2). Significant reductions of biofilm formation were obtained for all detected strains at all sub-MIC concentrations of *P*. *vera* L. ORS, except for *H*. *pylori* 3F/13 at 1/32 MIC. A similar trend was observed for LVX, in which a significant biofilm reduction was observed in all cases except for 1/32 MIC.

**Table 2:** Percentage reduction of *H*. *pylori* biofilm biomass (± SD) in presence of sub-MIC values of *P*. *vera* L. ORS and LVX.

% reduction of biofilm formation										
*H. pylori* strains	*P. vera* L. ORS					LVX				
	MIC					MIC				
	1/2	1/4	1/8	1/16	1/32	1/2	1/4	1/8	1/16	1/32
2A/12	90.80* ± 4.00	78.00* ± 7.40	75.20* ± 3.60	65.10* ± 6.70	44.10* ± 0.10	84.62* ± 0.20	76.26* ± 4.24	61.89* ± 3.39	43.14* ± 6.75	33.30 ± 7.94
10A/13	45.75* ± 1.18	39.15* ± 6.55	33.48* ± 1.36	33.44* ± 1.24	14.05* ± 2.26	50.92* ± 8.97	44.53* ± 2.03	37.48* ± 8.90	35.14* ± 6.05	25.95 ± 7.25
11F/11	81.17* ± 1.58	70.66* ± 7.67	66.17* ± 4.84	57.51* ± 1.38	42.42* ± 7.85	83.96* ± 4.40	66.92* ± 6.91	48.72* ± 9.92	40.59* ± 0.71	22.09 ± 2.12
3F/13	93.45* ± 4.34	82.88* ± 2.36	84.28* ± 0.57	51.63* ± 5.86	8.43 ± 1.79	64.52* ± 5.56	56.79* ± 5.05	39.80* ± 9.91	24.21* ± 0.78	23.07 ± 0.03
9F/13	76.06* ± 3.75	60.89* ± 9.18	49.51* ± 5.20	42.62* ± 2.85	38.49* ± 8.66	72.49* ± 4.44	65.40* ± 3.16	62.58* ± 2.60	50.93* ± 0.55	48.79 ± 2.49
ATCC 43629	77.90* ± 3.77	76.21* ± 5.54	71.68* ± 3.28	65.61* ± 6.30	34.68* ± 4.79	91.98* ± 0.38	86.40* ± 1.85	48.44* ± 3.18	41.93* ± 4.74	34.68 ± 4.79

*Statistically significant in respect to the control.

**Table 3:** Percentage reduction of *H*. *pylori* biofilm biomass (± SD) in presence of all sub-synergistic concentrations (FIC I ≤ 0.5) of *P*. *vera* L.ORS (mg/l) and LVX (mg/l).

% reduction of biofilm formation					
*H*. *pylori* strains (synergistic concentrations of ORS and LVX)	Synergistic concentrations				
	1/2	1/4	1/8	1/16	1/32
**2A/12**					
(390 + 0.12)	60.45* ± 5.47	51.57* ± 0.50	50.30* ± 1.84	22.49* ± 9.40	19.71* ± 4.17
(190 + 0.12)	58.63* ± 8.75	42.98* ± 7.12	37.02* ± 1.05	28.92* ± 2.68	25.62* ± 4.81
**10A/13**					
(390 + 0.25)	52.38* ± 2.06	6.41* ± 7.92	42.04* ± 4.98	30.08* ± 1.27	19.90 ± 9.66
**11F/11**					
(390 + 0.25)	37.77* ± 0.72	16.83* ± 2.45	16.80* ± 2.66	13.41* ± 3.45	0.00 ± 0.00
(390 + 0.12)	48.76* ± 2.16	21.53* ± 0.42	14.97* ± 6.70	12.99 ± 8.44	0.00 ± 0.00
(190 + 0.25)	60.19* ± 0.02	47.97* ± 0.37	39.04* ± 2.31	33.03* ± 2.70	31.75* ± 3.63
(190 + 0.12)	55.16* ± 1.81	52.05* ± 5.31	48.71* ± 5.95	10.38 ± 8.31	6.53 ± 3.41
(90 + 0.12)	54.06* ± 2.17	34.04* ± 0.60	6.69 ± 0.69	5.64 ± 0.57	1.72 ± 0.31
(90 + 0.25)	59.87* ± 0.30	56.03* ± 0.66	51.11* ± 1.33	50.93* ± 2.39	31.75* ± 3.63
**3F/13**					
(190 + 0.12)	50.90* ± 2.00	42.80* ± 3.20	0.10 ± 0.00	0.00 ± 0.00	0.00 ± 0.00
**9F/13**					
(390 + 0.03)	60.22* ± 5.74	55.65* ± 1.33	45.74* ± 4.70	33.30* ± 8.76	30.74* ± 8.63

^*^Statistically significant in respect to the control.

With regard to the anti-biofilm effect of sub-synergistic concentrations (Table 3), significant biofilm reductions, in respect to the controls, were obtained at 1/2, 1/4 and 1/8 synergistic concentrations for all detected strains except for *H*. *pylori* 11F/11 at 1/8 synergistic concentration (90 mg/l ORS + 0.12 mg/l LVX) and for *H*. *pylori* 3F/13 at 1/8 synergistic concentration. However, the major percentage of biofilm reduction was detected for the MDR *H*. *pylori* 2 A/12 showing a reduction rate of 60.45% ± 5.47 at 1/2 synergistic concentration (390 mg/l ORS + 0.12 mg/l LVX).

*Pistacia vera* L. ORS alone and combined with LVX displayed a significant *H*. *pylori* anti-biofilm activity.

**Figure 2:**
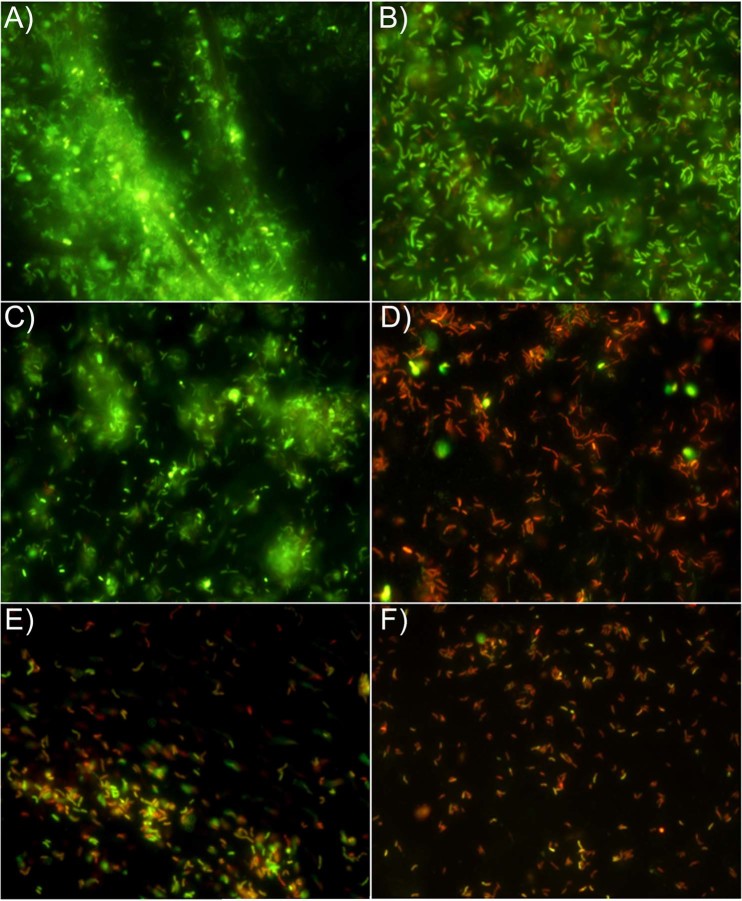
Representative images of Live/Dead staining of *H*. *pylori* 11F/11 biofilms. (**A**,**B**), control images (viable cells embedded in abundant biofilm were highlighted in **A** and viable rod shaped cells in the biofilm were highlighted in **B**). (**C**), biofilm treated with 1/4 MIC of *P*. *vera* L. ORS (prevalent viable coccoid cells in small aggregates). (**D**), biofilm treated with 1/8 MIC of LVX (prevalent dead rod cells without considerable aggregation). (**E**,**F**), biofilm treated with sub-synergistic concentrations of *P*. *vera* L. ORS and LVX showing their combined anti-adhesive and killing effect. (**E**), biofilm treated with 1/8 synergistic concentration of *P*. *vera* L. ORS (190 mg/l) and LVX (0.12 mg/l). (**F**), biofilm treated with 1/16 synergistic concentration of *P*. *vera* L. ORS (90 mg/l) and LVX (0.25 mg/l). Sessile population in biofilms stained in red (Propidium iodide) shows a damaged membrane (dead cells), whereas green stained bacteria (SYTO 9) represent viable cells. The images observed at fluorescent Leica 4000 DM microscopy (Leica Microsystems, Milan, Italy) were recorded at an emission wave length of 500 nm for SYTO 9 and of 635 nm for Propidium iodide and more fields of view randomly were examined. Original magnification, 1000X.

The Live/Dead assay was performed to evaluate the *H*. *pylori* viability showing also its capability to clusterize in presence of sub-MICs and sub-synergistic concentrations of *P*. *vera* L. ORS and LVX. A general reduction of *H*. *pylori* adhesion was observed in presence of all assayed sub-MIC concentrations of *P*. *vera* L. ORS and LVX as well as a marked loss of cell viability in presence of LVX. Figure 2 shows representative Live/Dead images of these effects in the resistant *H*. *pylori* 11F/11. In respect to the control (Fig. 2A,B), *P*. *vera* L. ORS reduced the *H*. *pylori* aggregation with viable and prevalent coccoid bacteria (Fig. 2C), instead, a marked killing action together with anti-adhesive effect were detected in presence of LVX (Fig. 2D). This effect was visualized by few adhering cells on polystyrene surface organized in small clusters of red cells. A combined anti-adhesive and killing action was detected in presence of sub-synergistic concentrations of *P*. *vera* L. ORS and LVX (Fig. 2E,F).

The toxicity of *P*. *vera* L. ORS at different doses was evaluated with the *in vivo G*. *mellonella* model. The survival percentage of *G*. *mellonella* at 1000 mg/kg of *P*. *vera* L. ORS was 80% after 1 day and 60% after 5 days. At doses lower than 1000 mg/kg, analogous data was obtained with a survival rate of 80–100%, that was similar to those obtained with PBS injection; therefore *P*. *vera* L. ORS can be deemed not toxic (Supplementary Fig. S1). During the experiments, *G*. *mellonella* control group was remained alive.

**Figure 3:**
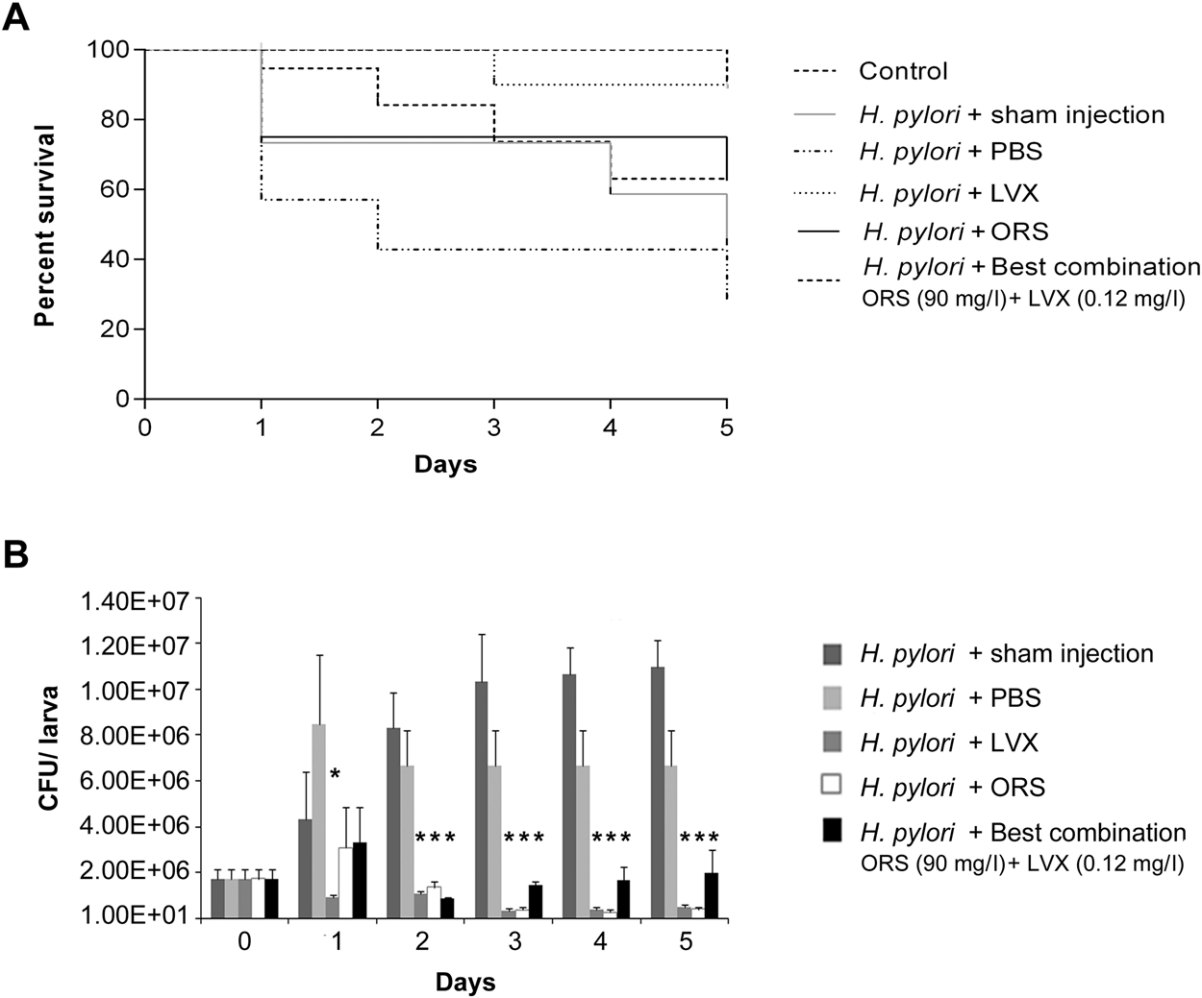
*In vivo* infection assay in *Galleria mellonella* model. (**A**) Survival of *G*. *mellonella* after *H*. *pylori* 11F/11 infection with PBS, MIC of LVX, 1000 mg/kg of *P*. *vera* L. ORS and the best synergistic combination of *P*. *vera* L. ORS and LVX (90 mg/l ORS + 0.12 mg/l LVX, FIC I = 0.18). Kaplan-Meier survival curves of *G*. *mellonella* larvae after infection with 1.8 × 10^6^ CFUs. Differences in survival were calculated using the Long-rank test for multiple comparison. (**B**) Recovery of *H*. *pylori* 11F/11 CFU/larva in *G*. *mellonella* larvae after injection of 1.8 × 10^6^ CFUs after different time points and at different conditions (PBS, LVX at MIC value, *P*. *vera* L. ORS at 1000 mg/kg, the best synergistic combination of *P*. *vera* L. ORS and LVX).*Statistically significant in respect to sham injection, at each time control (*p* ≤ 0.05).

In the *in vivo* infection assay, the survival of *G*. *mellonella* larvae treated with *H*. *pylori* 11F/11 and LVX at MIC concentration, *P*. *vera* L. ORS at 1000 mg/kg, and the best combination of *P*. *vera* L. ORS and LVX (90 mg/l ORS + 0.12 mg/l LVX) was checked everyday until 5 days. The treatment with LVX rescued larvae injected with *H*. *pylori* with a survival rate between 90% and 100%. The treatment with *P*. *vera* L. ORS rescued larvae from *H*. *pylori* infection by 75% until 4 days and by 62% at 5 days. The best synergistic combination of *P*. *vera* L. ORS plus LVX showed a protective effect against *H*. *pylori* infection with larvae survival rate of 90% after 1 day and 63% after 5 days (Fig. 3A). The differences were compared with Long-rank test and the survival curves were statistically significant (*p* = 0.0008).

*Pistacia vera* L. ORS, both alone and combined with LVX, showed a protective effect against *H*. *pylori* infection over time.

The ability of *H*. *pylori* suspension of 1.8 × 10^6^ CFUs to infect *G*. *mellonella* larvae was analyzed at different times, by CFU determination. As shown in Fig. 3B, *H*. *pylori* 11F/11 was able to infect *G*. *mellonella* larvae and to grow over time. In the presence of LVX, the high survival rate of *G*. *mellonella* larvae is related to the reduced *H*. *pylori* survival rate, demonstrated by the low CFU values detected after 1, 2, 3, 4 and 5 days (*p* ≤ 0.05). *Pistacia vera* L. ORS confirms its protective effect in *G*. *mellonella* larvae reducing significantly the bacterial load starting from 2 days (*p* ≤ 0.05). In presence of the best synergistic combination of *P*. *vera* L. ORS plus LVX, a significant *H*. *pylori* reduction was detected after 2 days (*p* ≤ 0.05) (Fig. 3B).

*Pistacia vera* L. ORS exhibits its anti-*H*. *pylori* effect also in *in vivo* model.

## Discussion

In this study, the antimicrobial and anti-biofilm activities of *P*. *vera* L. ORS alone and combined with LVX against resistant clinical *H*. *pylori* strains were evaluated.

Antibiotic resistance is presently a remarkable issue and, recently, a great number of studies suggest that the addition of medicinal plants to standard treatments, commonly used in therapy could emphasize the eradication rate of *H*. *pylori*^9,13,22,28^.

Our data shows an antimicrobial effect of *P*. *vera* L. ORS against resistant *H*. *pylori* strains both in neutral and acidic environments suggesting its potential inclusion in treatments of *H*. *pylori* related gastric infections. In particular, the real goal of this study is the evaluation of the combined action of *P*. *vera* L. ORS and LVX that synergize to each other restoring the antimicrobial drug efficacy. *Pistacia vera* L. ORS is able to restore the effectiveness of LVX by reducing its MIC values under the breakpoint against MDR and resistant *H*. *pylori* strains.

Safavi *et al*.^13^ demonstrated that the antibacterial action of *P*. *vera* L. was correlated to the acid fraction containing terpenes. Another possible mechanism of antimicrobial activity of *P*. *vera* L. ORS could be related to its volatile components. In fact, ORS is characterized by a percentage of volatile monoterpenes of 15–30% which make it more fluid than the resins properly so-called. This ORS characteristic could favor the diffusion of antibiotics across bacterial membranes and/or hinder the efflux pumps that are a common resistance mechanism in Gram-negative bacteria^29^. Mechanisms of the pharmacological synergism are related to the multi-action in different target sites in the bacterial cell, pharmacokinetic or physicochemical effects (e.g. improvement of solubility or bioavailability) and/or action on bacterial resistance mechanism^30,31^. As well known, *P*. *vera* L. induces protrusions, morphological abnormalities and cellular fragmentation in *H*. *pylori* cells and probably facilitates the penetration and killing action of LVX^11,22^.

The anti-biofilm properties of *P*. *vera* L. ORS alone and in association with LVX were also evaluated on *H*. *pylori* biofilm formation. *Pistacia vera* L. ORS exhibits a strong anti-biofilm activity. The resin, a product of the *Pistacia* genus, contains a high molecular weight polymer *cis*-1,4-poly-β-myrcene, that shows antimicrobial and anti-adhesive actions^32^. Sharif *et al*.^33^ showed that this natural compound exhibited both anti-plaque activity and *H*. *pylori* growth reduction. In our study, each sub-synergistic concentration displayed an important anti-biofilm effect. In particular, a stronger anti-adhesive effect was more evident when in the synergistic combinations *P*. *vera* L. ORS prevailed *vs* LVX, and a remarkable killing effect when LVX prevailed *vs P*. *vera* L. ORS.

*Galleria mellonella* is a validated *in vivo* model chosen for studies of pathogenesis of bacterial or fungal infections and it realizes the similar condition of disease development in the human body. Moreover, *G*. *mellonella* is used to study the toxicity and the efficacy of antibiotics and antifungal agents. Giannuoli *et al*.^34^ showed that *G*. *mellonella* larvae are susceptible to *H*. *pylori* infection and it is a good *in vivo* model to evaluate the virulence factors and pathogenic mechanisms of *H*. *pylori*^34,35^. In some studies, the *in vitro* activity of natural products did not correlate with *in vivo* data due to the lack of specificity action of the molecule^25^. Here, we infected *G*. *mellonella* with the resistant *H*. *pylori* 11F/11 to evaluate the antimicrobial efficacy of *P*. *vera* L. ORS observing a protective effect against *H*. *pylori* infection, confirming the *in vitro* data. Levofloxacin, at MIC concentration, reduces significantly the *H*. *pylori* replication in *G*. *mellonella* larvae with a high percentage of survival larvae. Interestingly, *P*. *vera* L. ORS alone or combined with LVX shows a significant protective effect and this remarkable data suggests its potential use in *H*. *pylori* infection.

*Pistacia vera* L. ORS is a phyto-complex able to restore the effectiveness of LVX by reducing its MICs. The co-administration with the antibiotics commonly used in therapy could improve the potency of the treatment by repositioning certain marketed antimicrobial compounds. In this way, the use of natural compounds can overcome many of the commercial barriers regarding the developing of new antibiotics restoring the activity of ineffective drugs^16^.

Overall, our findings underline that the use of this natural compound, combined to LVX, could represents an effective and innovative strategy to tackle the antibiotic resistance and biofilm forming capability in *H*. *pylori*.

To the best of our knowledge, this is the first work that shows the effect of *P*. *vera* L. ORS against *H*. *pylori* strains with remarkable results relative to its antimicrobial and anti-biofilm effects. Future studies will be required to better investigate both the effect of this phyto-complex combined with other antimicrobials used in *H*. *pylori* infection and the antimicrobial efficacy of single active compounds of *P*. *vera* L. ORS.

## Materials and Methods

### *Pistacia vera* L. ORS recovery and chemical fingerprinting

*Pistacia vera* L. raw ORS was obtained by making incisions from the base of the trunk of plants from a private pistachio garden in Bronte (Catania, Sicily, Italy)^36^. α-pinene, β-pinene, and alkanes standard solutions C_8_-C_20_ were purchased from Sigma-Aldrich (USA), pure standards were purchased from Labochem (Italy), Chemfaces (China) and Extrasynthese (France). The raw ORS was filtered with 100 ml of diethyl ether on filter paper (Whatman, cat. n° 1004-930, grade 4). The volume was reduced gently under vacuum with a rotary evaporator until the filtrate was a yellow-amber crystalline solid. The ORS was fractioned to acidic and neutral fraction according to Barton and Seoane^37^. To improve its chromatographic behavior, as reported by Assimopoulou and Papageorgiou^38,39^, the acidic fraction was methylated under reflux with CH_3_I in presence of K_2_CO_3_ to obtain the methylated fraction used for analytical purposes only. The identity of the ORS components was established from its GC retention indices, relative to C_8_-C_20_ alkanes, by comparing their fragmentation patterns with those reported in the literature and by computer matching with the NIST MS 107, NIST 21 libraries, using the software GCMS solution version 1.02 (Lab solution, Shimatzu), by co-injection with authentic samples^40^.

### Bacterial cultures

For experiments, thirty resistant and MDR *H*. *pylori* clinical strains were used. All these strains were resistant to LVX (MICs ≥ 0.5 mg/l), MDR strains displayed the resistance at least three antimicrobial classes. The susceptible clinical strain *H*. *pylori* 9F/13 and the reference strain *H*. *pylori* ATCC 43629 were also included. The used strains together with their susceptible panel to antibiotics were listed in Supplementary Table S1. The bacteria were cultured on chocolate agar containing Columbia agar base (CA, Oxoid, Milan, Italy) with 10% (v/v) lacked horse blood plus IsoVitalex 1% (v/v) (BBL, Microbiology System, Milan, Italy) and stored at −80 °C. The bacterial suspension were prepared in Brucella Broth (BB) plus fetal calf serum 2% (FS) (Biolife Italiana, Milan, Italy) and adjusted to an optical density at 600 nm (OD_600_) of 0.2 (1.8 × 10^7^ CFU/ml approximately), by using a Biophotometer (Eppendorf, Milan, Italy)^21^.

### Antibacterial susceptibility assay

The determination of *P*. *vera* L. ORS and LVX MICs against standardized broth culture of *H*. *pylori* strains (prepared as describe above) was determined with broth microdilution method in 96-wells-microtitre plates^41^ (Nunc, Euro Clone SpA, Life Sciences-Division, Milan, Italy). Twofold dilutions of *P*. *vera* L. ORS stock solution ranging from 6250 to 780 mg/l were performed in BB plus FS. Levofloxacin (Sigma Aldrich S.R.L, Milan, Italy) was prepared in BB plus FS in twofold dilutions from 2.00 to 0.01 mg/l. One hundred µl of *P*. *vera* L. ORS or 100 µl LVX and 100 μl standardized bacterial suspension were dispensed in each well of 96-wells-microtitre plate and incubated in microaerobic condition for 3 days at 37 °C. MIC values were measured by determining the lowest concentration of *P*. *vera* L. ORS and LVX able to inhibit the visible growth of the microorganisms. MBCs were determined by sub-culturing 10 μl of suspensions from the non-turbid wells on CA and incubated as describe above. The MBC represents the lowest concentration of *P*. *vera* L. ORS or LVX that inhibited the bacterial growth on plates. To evaluate a possible effect of pH variation on *P*. *vera* L. ORS, the MICs and MBCs were also determined in acid conditions by aseptically adding adequate amounts of 1N HCl to the medium to achieve a final pH value 5.5.

### Checkerboard assay

Antibacterial synergisms between *P*. *vera* L. ORS and LVX was determined by checkerboard test. Dilutions of the two substances, from MICs to serial dilution below, were inoculated in microtiter plates and incubated as described above^15^. The checkerboard test was used to calculate the Fractional Inhibitory Concentration (FIC), that is equal to MICAB/MICA + MICBA/MICB, where MICAB is the MIC of compound A in presence of compound B; MICBA is MIC of B in presence of A. FIC Index (FIC I) values were interpreted according to Odds^42^ namely synergism FIC I ≤ 0.5, antagonism FIC I ≥ 4.0, and additive FIC I > 0.5–4.0. For the control, *P*. *vera* L. ORS and LVX were assayed alone. The results were also reported as isobolograms constructed by plotting synergistic concentrations^43^.

### Biofilm biomass quantification and cell viability analysis

Anti-biofilm activity of *P*. *vera* L. ORS, LVX (at sub-MICs) and all their synergistic concentrations^44^ (FIC I ≤ 0.5, at sub-synergistic concentrations) were evaluated on the biofilm formation of two representative MDR (2A/12, 10A/13), two resistant (11F/11, 3F/13), one susceptible (9F/13) and the reference ATCC 43629 strains. Broth cultures of *H*. *pylori*, harvested in BB supplemented with 2% (w/v) SF (Biolife, Italy) and 0.3% (w/v) glucose, were gently shaken and incubated overnight at 37 °C in a microaerobic atmosphere. After incubation, each broth culture was adjusted to OD_600_ = 0.1 and 100 µl of standardized broth culture was inoculated on flat-bottomed 96-wells-polystyrene- microtiter plates with *P*. *vera* L. ORS (100 µl), LVX (100 µl) or with sub-synergistic concentrations (50 μl of each substance). After incubation at 37 °C in microaerobic atmosphere for 48 hours, the produced biomasses of the treated and untreated biofilms were determined by safranin staining method^21^.

For the evaluation of cell viability, biofilms were grown in presence of sub-MIC concentrations of *P*. *vera* L. ORS, LVX and with all sub-synergistic combinations. Briefly, 1 ml of *P*. *vera* L. ORS or 1 ml of LVX or 1 ml (500 µl + 500 µl) of the all sub-synergistic combinations and 1 ml of standardized broth cultures of *H*. *pylori* 11F/11 were inoculated in Petri dish (3.5 cm) and incubated as describe above. After incubation, the planktonic cells were removed from each Petri dish and the sessile bacterial populations were washed with PBS and stained with Backlight Live/Dead Viability staining (Molecular Probes, Invitrogen detection technologies, USA) as indicated by manufacturer^45^. The images were observed at fluorescent Leica 4000 DM microscopy (Leica Microsystems, Milan, Italy), and more fields of view were examined randomly.

### *Pistacia vera* L. ORS toxicity test

The toxicity was evaluated by using wax moth *G*. *mellonella* larvae. Stock solution of *P*. *vera* L. ORS was diluted in PBS to obtain the following twofold final concentrations: 1000, 500, 250, 125 and 62.5 mg/kg. Seven groups of ten randomly-selected *G*. *mellonella* larvae, weighing 0.2–0.3 g, were treated as follows: five groups were injected in the last left proleg with 10 μl of each concentration of *P*. *vera* L. ORS and one group was injected with PBS by using Hamilton syringe, one group was uninjected. A total of seventy larvae were incubated at 37 °C in Petri dishes in dark for 5 days. The *G*. *mellonella* survival was evaluated every 24 hours; larvae were considered dead when were unresponsive to touch^46^. During assays, larvae did not receive nutrition.

### *In vivo G*. *mellonella* infection assay

The *in vivo* activity of *P*. *vera* L. ORS against *H*. *pylori* was evaluated by using *G*. *mellonella* larvae, that represents a recognized model for *H*. *pylori* infection^34^ and does not require ethical approval. *H*. *pylori* 11F/11, standardized at OD_600_ = 0.25 (1.8 × 10^6^ CFU/ml), was the clinical strain used for experiments. Five groups of ten randomly selected *G*. *mellonella* larvae were injected with 10 μl of *H*. *pylori* suspension, in the last left proleg of each larva for a total of fifty larvae. After two hours, ten larvae were treated with 10 μl of LVX at MIC value, ten larvae were treated with *P*. *vera* L. ORS at 1000 mg/kg, ten larvae were treated with the best synergistic combination of *P*. *vera* L. ORS plus LVX (90 mg/l ORS + 0.12 mg/l LVX) on the last right proleg. A control group of ten larvae was treated with 10 μl of PBS and ten larvae with a sham injection. For sham injection, the larvae were nicked with the syringe to evaluate the effect of the larval puncture. Larvae were incubated at 37 °C in Petri dishes in dark for 5 days. The *G*. *mellonella* survival was evaluated everyday; larvae were considered dead when unresponsive to touch.

To determine the survival rate of bacteria in larvae after 1, 2, 3, 4 and 5 days post-infection, fifty larvae were infected with 10 μl of *H*. *pylori* suspension (1.8 × 10^6^ CFU/ml) and after two hours, ten larvae were treated with 10 μl of LVX at MIC values, ten larvae were treated with *P*. *vera* L. ORS at 1000 mg/kg_,_ ten larvae were treated with the best synergistic combination of *P*. *vera* L. ORS and LVX, ten larvae was treated with PBS and ten larvae received a sham injection as describe above. After incubation, three larvae, for each group, were chilled on ice for 10 min, aseptically removed and the haemocoel was drained into a sterile 1.5 ml Eppendorf tube. Haemocoel was serially diluted in PBS and the bacterial cells were quantified by enumeration of CFUs on Campylobacter selective agar (CP Dent) with 7% defibrinated horse blood and 0.4% of Dent supplement (Oxoid) and incubated under microaerobic condition at 37 °C. The CFU/larva were counted after 3 days. During assays, larvae did not receive nutrition.

### Statistical analysis

Data is obtained from at least three independent experiments performed in triplicate. Data is shown as the means ± standard deviation (SD). Differences between groups were assessed with paired Student’s t-test. *P* values ≤ 0.05 were considered statistically significant. Survival curves were plotted using the Kaplan-Meier method, and survival differences were calculated using the Long-rank test for multiple comparisons. GraphPad Prism 6 was used to fit a curve to the infection data.

## Supplementary information

Supplementary Information

## References

[1] Graham DY (2009). Efficient identification and evaluation of *effective Helicobacter pylori* therapies. Clin Gastroenterol Hepatol.

[2] Mégraud F, Lehours P (2007). Helicobacter pylori detection and antimicrobial susceptibility testing. Clin Microbiol Rev.

[3] Vagarali MA (2015). Clinical significance of various diagnostic techniques and emerging antimicrobial resistance pattern of Helicobacter pylori from gastric biopsy samples. Indian J Med Microbiol.

[4] Cellini L (2014). Helicobacter pylori: a chameleon-like approach to life. World J Gastroenterol.

[5] Cellini L (2005). Biofilm formation and modulation of luxS and rpoD expression by Helicobacter pylori. Biofilms.

[6] Cellini L (2008). Dynamic colonization of Helicobacter pylori in human gastric mucosa. Scand J Gastroenterol.

[7] Perez Aldana L (2002). The relationship between consumption of antimicrobial agents and the prevalence of primary Helicobacter pylori resistance. Helicobacter.

[8] Megraud F (2013). Study Group participants. Helicobacter pylori resistance to antibiotics in Europe and its relationship to antibiotic consumption. Gut.

[9] De Francesco, V. *et al*. Worldwide H. pylori antibiotic resistance: a systematic review. *J Gastrointest Liver Dis* **19**, 409–414, PMID: 21188333 (2010). 21188333

[10] Di Giulio M (2016). *In vitro* antimicrobial susceptibility of Helicobacter pylori to nine antibiotics currently used in Central Italy. Scand J Gastroenterol.

[11] Shmuely H, Domniz N, Yahav J (2016). Non-pharmacological treatment of Helicobacter pylori. World J Gastrointest Pharmacol Ther.

[12] Ayala G (2014). Exploring alternative treatments for Helicobacter pylori infection. World J Gastroenterol.

[13] Safavi M, Shams-Ardakani M, Foroumadi A (2015). Medicinal plants in the treatment of Helicobacter pylori infections. Pharm Biol.

[14] Topuzovic MD (2015). Phytomedical investigation of Najas minor All. in the view of the chemical constituents. EXCLI J.

[15] Marini E (2018). Curcumin, an antibiotic resistance breaker against a multiresistant clinical isolate of Mycobacterium abscessus. Phytother Res.

[16] Brown D (2015). Antibiotic resistance breakers: Can repurposed drugs fill the antibiotic discovery void?. Nat Rev Drug Dis.

[17] Lyddiard D, Jones GL, Greatrex BW (2016). Keeping it simple: lessons from the golden era of antibiotic discovery. FEMS Microbiol Lett.

[18] Bozorgi M (2013). Five Pistacia species (*P. vera, P. atlantica, P. terebinthus, P. khinjuk*, and *P. lentiscus*): a review of their traditional uses, phytochemistry, and pharmacology. Sci World J.

[19] Fazeli-Nasab B, Fooladvand Z (2016). A Review on Iranian Carumcopticum (L.): Composition and Biological Activities. Europ J Med Plants.

[20] Nostro A (2005). Antibacterial effect of plant extracts against Helicobacter pylori. Phytother Res.

[21] Cataldi V (2015). *In vitro* activity of Aloe vera inner gel against microorganisms grown in planktonic and sessile phases. Int J Immunopathol Pharmacol.

[22] Marone P (2001). Bactericidal activity of Pistacia lentiscus mastic gum against Helicobacter pylori. J Chemother.

[23] Dabos KJ (2010). The effect of mastic gum on Helicobacter pylori: a randomized pilot study. Phytomedicine.

[24] Miyamoto T, Okimoto T, Kuwano M (2014). Chemical Composition of the Essential Oil of Mastic Gum and their Antibacterial Activity against Drug-Resistant Helicobacter pylori. Nat Prod Bioprospec.

[25] Debraekeleer A, Remaut H (2018). Future perspective for potential Helicobacter pylori eradication therapies. Published online:. Future Microbiol.

[26] Seyyedmajidi M (2016). Addition of cranberry to proton pump inhibitor-based triple therapy for Helicobacter pylori eradication. J Res Phar Pract.

[27] Ciccaglione, A. F. *et al*. Bovine lactoferrin enhances the efficacy of levofloxacin based triple therapy as first line treatment of Helicobacter pylori infection: an in vitro and in vivo study. In press in *J Antimicrob Chemother* (2018). 10.1093/jac/dky510 PMC6419617 30668729

[28] Maheshwari, M. *et al*. Bioactive extracts of Carumcopticum L. enhances efficacy of ciprofloxacin against MDR enteric bacteria. *Saudi J Biol Sci* (article in press), 10.1016/j.sjbs.2017.12.008 (2018). 10.1016/j.sjbs.2017.12.008 PMC6864163 31762667

[29] Bolla JM (2011). Strategies for bypassing the membrane barrier in multidrug resistant Gram-negative bacteria. FEBS Lett.

[30] Hemaiswarya S, Kruthiventi AK, Doble M (2008). Synergism between natural products and antibiotics against infectious diseases. Phytomedicine.

[31] Wagner H, Ulrich-Merzenich G (2009). Synergy research: approaching a new generation of phytopharmaceuticals. Phytomedicine.

[32] Sharifi MS (2011). Bio-activity of natural polymers from the genus Pistacia: a validated model for their antimicrobial action. Global J Health Sci.

[33] Sharifi MS, Hazell SL (2009). Fractionation of Mastic Gum in Relation to Antimicrobial Activity. Pharmaceuticals (Basel).

[34] Giannouli M (2014). Use of larvae of the wax moth Galleria mellonella as an *in vivo* model to study the virulence of Helicobacter pylori. BMC Microbiol.

[35] Mikulak, E. *et al*. Galleria mellonella *L*. as model organism used in biomedical and other studies. *Przegl Epidemiol* **72**, 57–73, PMID:29667381 (2018). 29667381

[36] Magi G (2018). Chemical composition of Pistacia vera L. oleoresin and its antibacterial, anti-virulence and anti-biofilm activities against oral streptococci, including Streptococcus mutans. Arch Oral Biol.

[37] Barton DHR, Seoane E (1956). Triterpenoids. Part XXII. The constitution and stereochemistry of masticadienonic acid. J Chem Soc.

[38] Assimopoulou AN, Papageorgiou VP (2005). GC-MS analysis of penta- and tetra-cyclic *triterpenes* from resins of *Pistacia* species. Part I. *Pistacia lentiscus* var. Chia. Biomed Chromatogr.

[39] Assimopoulou AN, Papageorgiou VP (2005). GC-MS analysis of penta- and tetra-cyclic triterpenes from resins of Pistacia species. Part II. *Pistacia terebinthus* var. Chia. Biomed Chromatogr.

[40] Adams, R. P. Identification of essential oil components by gas chromatography/mass spectrometry, 4th edition. Allured Publishing Corp., Carol Stream, IL, USA (2007).

[41] Clinical and Laboratory Standards Institute. CLSI. 2018. Methods for dilution antimicrobialsusceptibility tests for bacteria that grow aerobically, 11th Edition, M07-Ed11 and M100-Ed28 Package of 2 Docs Wayne, PA (2018).

[42] Odds FC (2003). Synergy, antagonism, and what the checkerboard puts between them. J Antimicrob Chemother.

[43] Mulyaningsi S (2010). Synergistic properties of the terpenoids aromadendrene and1,8‐cineole from the essential oil of Eucalyptus globulus against antibiotic‐susceptible and antibiotic‐resistant pathogens. Phytomedicine.

[44] Gonzalez Moreno M, Trampuz A, Di Luca M (2017). Synergistic antibiotic activity against planktonic and biofilm-embedded Streptococcus agalactiae, Streptococcus pyogenes and Streptococcus oralis. J Antimicrob Chemother.

[45] Di Lodovico S (2017). Enterococcus hirae biofilm formation on hospital material surfaces and effect of new biocides. Environ Health Prev Med.

[46] Tharmalingam N (2018). Repurposing the anthelmintic drug niclosamide to combat Helicobacter pylori. Sci Reports.

