# Supplementary material for: Pistacia vera L. oleoresin and levofloxacin is a synergistic combination against resistant Helicobacter pylori strains
Source: Sci Rep. 2019 Mar 15;9:4646. doi: 10.1038/s41598-019-40991-y (PMC6420558; doi:10.1038/s41598-019-40991-y)
Supplement: Supplementary file 1 — Supplementary Information [file 41598_2019_40991_MOESM1_ESM.docx]

**Supplementary Information**

***Pistacia vera* L. oleoresin and levofloxacin is a synergistic combination against resistant *Helicobacter pylori* strains**

**Silvia Di Lodovico^1^, Edoardo Napoli^2^, Emanuela Di Campli^1^, Paola Di Fermo^1^, Davide Gentile^2^, Giuseppe Ruberto^2^, Antonia Nostro^3^, Emanuela Marini^4^, Luigina Cellini^1^& Mara Di Giulio^1^***

^1^Department of Pharmacy, “G. d’Annunzio” University Chieti-Pescara, Chieti, Italy. ^2^Institute of Biomolecular Chemistry, National Research Council ICB-CNR, Catania, Italy. ^3^Department of Chemical, Biological, Pharmaceutical and Environmental Sciences, University of Messina, Messina, Italy. ^4^Unit of Microbiology, Department of Biomedical Sciences and Public Health, Polytechnic University of Marche, Ancona, Italy.

*Correspondence should be addressed to M.D.G. (email: [mara.digiulio@unich.it](mailto:mara.digiulio@unich.it)) Tel: +390871 3554579, Fax: +390871 3554562

Table S1 Antimicrobial susceptibility panel of *H. pylori* clinical strains used in this study

| *H. pylori* | Antimicrobials* | | | | | | | | |  |
| --- | --- | --- | --- | --- | --- | --- | --- | --- | --- | --- |
|  | CLA | MET | LVX | MOXI | CIP | RIF | TET | AMP | AMOX |  |
| 11F/11 | R | S | R | R | R | S | S | S | S |  |
| 2A/12** | R | R | R | R | R | S | S | S | S |  |
| 3F/12 | R | S | R | R | R | S | S | S | S |  |
| 4A/12 | R | S | R | S | S | S | S | S | S |  |
| 7A/12 | S | S | R | R | R | S | S | S | S |  |
| 9A/12 | R | S | R | R | R | S | S | S | S |  |
| 12F/12 | R | S | R | R | R | S | S | S | S |  |
| 13A/12 | R | S | R | R | R | S | S | S | S |  |
| 1F/13 | S | S | R | R | S | S | S | S | S |  |
| 3F/13 | R | S | R | R | R | S | S | S | S |  |
| 4A/13** | R | R | R | R | R | S | S | S | S |  |
| 5A/13** | R | R | R | R | R | S | S | S | S |  |
| 10A/13** | R | R | R | R | R | S | S | S | S |  |
| 13A/13** | R | R | R | R | R | S | S | S | S |  |
| 20A/13 | R | S | R | R | S | S | S | S | S |  |
| 23A/13 | R | S | R | R | R | S | S | S | S |  |
| 24F/13 | R | S | R | R | R | S | S | S | S |  |
| 25F/13 | R | S | R | R | R | S | S | S | S |  |
| 26A/13** | R | R | R | R | R | S | S | S | S |  |
| 5F/14 | S | S | R | R | R | S | S | S | S |  |
| 10A/14** | R | R | R | R | R | S | S | S | S |  |
| 29A/14 | R | S | R | R | R | S | S | S | S |  |
| 3F/15 | R | S | R | R | R | S | S | S | S |  |
| 4A/15 | S | S | R | R | R | S | S | S | S |  |
| 8F/15 | R | S | R | R | R | S | S | S | S |  |
| 30A/15** | R | R | R | R | R | S | S | S | S |  |
| 1A/16 | R | S | R | R | R | S | S | S | S |  |
| 5A/16 | R | S | R | R | R | S | S | S | S |  |
| 7F/16** | R | R | R | R | R | S | S | S | S |  |
| 14A/16 | R | S | R | S | S | S | S | S | S |  |
| 9F/13 | S | S | S | S | S | S | S | S | S |  |
| ATCC 43629 | S | S | S | S | S | S | S | S | S |  |

*Abbreviations: CLA, clarithromycin; MET, metronidazole; LVX, levofloxacin, MOXI, moxifloxacin; CIP, ciprofloxacin; RIF, rifabutin; TET, tetracycline; AMP, ampicillin; AMOX, amoxicillin.

**Multidrug resistance (MDR) strains. MDR is defined as antimicrobial resistance shown by a strain with resistance at least three antimicrobial classes [1]

**Figure S1** *Pistacia vera* L. ORS toxicity in *Galleria mellonella* larvae at different concentrations (mg/kg)

**
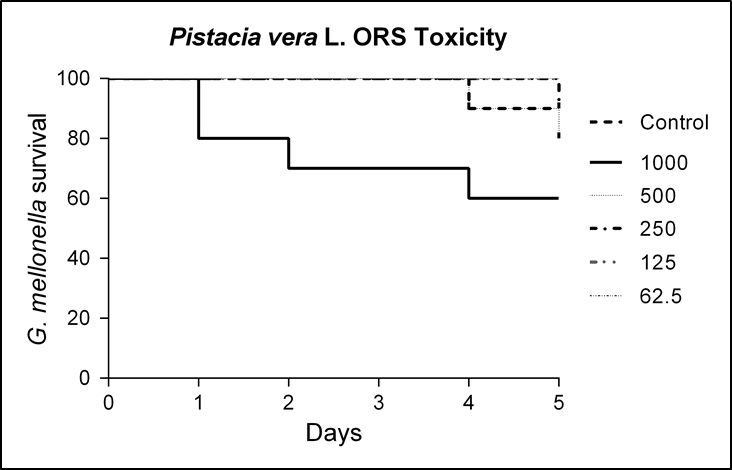
**

**Figure legend**

**Figure S1** *Pistacia vera* L. ORS toxicity assay in *Galleria mellonella* model. Kaplan-Meier survival curves of *G. mellonella* after treatment with 1000, 500, 250, 125, 62.5 mg/kg of *P. vera* L. ORS.

**References**

1. Magiorakos, A. P. *et al*. [Multidrug-resistant, extensively drug-resistant and pandrug-resistant bacteria: aninternational expert proposal for interim standard definitions for acquired resistance.](https://www.ncbi.nlm.nih.gov/pubmed/21793988) *Clin Microbiol Infect* 18(3), 268–281, http://doi:10.1111/j.1469-0691.2011.03570.x (2011).
